# Annotations

**Published:** 1897-02-13

**Authors:** 


### ANNOTATIONS.

### The Prevalence of Sleeplessness.

It is probable that most medical men, whose work lies largely among those who toil with their brains, have noticed the unusual prevalence of sleeplessness during the present winter, and more especially amongst men. Patient after patient repeats the same story. He goes to bed at his usual hour, falls off to sleep very much as usual, but, instead of sleeping through the whole night until six or seven in the morning, he wakes about three, or even earlier, and, do what he will, he can get "no sound sleep after that time." He may lie more or less still, and may even "doze off" occasionally; but if he does he dreams, or is more than half conscious, and in the morning when it is time to rise he feels, not only unrefreshed, but as if he would give all his day's profits for one single hour of sound refreshing sleep. But thas may not be. Now, there are three things to be said on this point—first, something as to the cause; secondly, as to the treatment to be avoided; and, thirdly, as to the treatment which will probably cure. The cause is, no doubt, the absence of clear, bright, frosty weather, and the prevalence of a damp, relaxing atmosphere of relatively high temperature for the season. That this is the true cause is practically proved by the improved sleep which most patients obtained during the few sharp frosty nights of a week or two ago. Under the circumstances what is to be done? One thing must certainly not be done; soporifics must not be resorted to. The right thing to do is, if possible, to diminish, or altogether stop, excessive brain activity. The most effectual step towards this end is to run away to the seaside for a few days or a week, and to a cold bracing place. To take sleep-producing remedies may answer the purpose for a short time; but such a course cannot but be attended with after injury under the peculiar physiological conditions. A few days of brain rest, and brain-bracing at the seaside will, with certainty, effect a "natural" cure in most cases, and the effect upon the whole system will be as lasting as it will be beneficial.

### The Extirpation of Tuberculosis.

Sir James Sawyer proposes to the profession and the public, through the medium of the *Times* newspaper, an energetic crusade against tuberculosis. This is a logical outcome of the definite knowledge which the profession now possesses on the subject. Sir James himself has been invited to attend an assemblage of scientists, practising physicians, agriculturists, and others at his own county town of Warwick, and to confer with them as to the most practicable steps to be taken for the total extirpation of the deadly microbe amongst agricultural animals, and the limitation of its ravages among the human kind. The problem to be solved may not perhaps be found as difficult in practice as some of us may be inclined to think. As Sir James Sawyer points out, "the tubercle bacillus is one and the same, whatever living animal being or animal tissue it may invade. Human beings are infected from each other and from the lower animals, and the lower animals from each other and from human beings. The reciprocal process goes on through manifold varieties of intercourse." Universal "stamping out" among men and animals is, of course, impossible; but the process may at least be applied to all sorts of animals. No doubt the cost of the earlier stages of a "stamping out" process will be enormous, by reason of the large number of animals which must necessarily be destroyed. But the slaughtering of tuberculous animals does not always mean their altogether profitless destruction. Large portions of the flesh of such animals in the earlier stages is available for human and animal food if properly cooked. If good is to be done, however, in this direction the movement must not be confined to Warwick or to a limited number of agricultural centres, but a whole series of such movements must be made to embrace and include the whole nation. County authorities would do well to secure the services of an adequate staff of competent young medical scientists, and to offer them such remuneration as will encourage them to take up county and imperial sanitary work, and to find in it a career worthy of their high and nationally serviceable scientific attainments. If the work is to be competently done, it will be indispensable to employ not only competent hands, but competent heads.

### Antiplague Serum.

M. Yersin, of the Pasteur Institute, will, we may hope, soon be at work in Bombay making critical experiments with an "antiplague" serum. There are now in a condition of readiness a large number of immunised animals. Experts from almost every civilised country are, or soon will be, on the spot in Bombay; and we shall soon have, as a contemporary writes, "a complete enquiry . . . conducted by experts of several nations, not holding briefs for particular policies . . . but free to concentrate their energies in an impartial investigation of the facts." The plague, it is felt, is a deplorable calamity. But it has at least this feature about it, that it offers an opportunity, unparalleled in modern times, for the acquisition of pathological knowledge and for trying on a gigantic scale a method of treatment, still novel, and either revolutionary in its possibilities, or of hardly any importance at all. We need not, perhaps, anticipate any universal prevalence of a disease like the plague; and, therefore, from that single point of view the serum treatment may not be held to be of anything but temporary interest. It is, however, from the standpoint, not of the serum treatment of plague alone, but of the treatment of acute specific diseases in general that the present unparalleled opportunity is so important and interesting. Scientific men realise that mere hypotheses, however plausible, can never take rank as scientific advances. Facts there must be; facts which are absolutely demonstrable; results which, under like conditions, always come out the same, whoever be the makers or the subjects of the experiments. In this country we look with expectant eyes towards the gigantic serum experiments which are now commencing. It is hardly, perhaps, too much to prophesy that if the plague continues for any considerable length of time the serum treatment will either be permanently established or much discredited in the result.

